# Incidental Diagnosis of Serous Tubal Intraepithelial Carcinoma During Infertility Treatment: A Case Report

**DOI:** 10.7759/cureus.96355

**Published:** 2025-11-08

**Authors:** Keisuke Doi, Yasuhiro Ohara, Tsuyoshi Takiuchi, Tomomoto Ishikawa

**Affiliations:** 1 Reproductive Medicine, Reproduction Clinic Osaka, Osaka, JPN; 2 Obstetrics and Gynecology, The University of Osaka, Osaka, JPN

**Keywords:** assisted reproductive technology (art), brca, hydrosalpinx, laparoscopic salpingectomy, serous tubal intraepithelial carcinoma

## Abstract

Serous tubal intraepithelial carcinoma (STIC) represents a precursor lesion to high-grade serous ovarian carcinoma and is typically identified during prophylactic surgery in BRCA mutation carriers. To the best of our knowledge, we report the first case of STIC incidentally diagnosed during infertility treatment. A 43-year-old nulligravid woman with four years of primary infertility underwent laparoscopic left salpingectomy for hydrosalpinx prior to embryo transfer. Histopathological examination unexpectedly revealed STIC, confirmed by characteristic morphological features and immunohistochemical markers, including strong nuclear p53 expression and elevated Ki-67 index. The patient declined BRCA genetic testing and prophylactic surgical interventions, opting to continue fertility treatment under enhanced surveillance. This unprecedented case emphasizes the critical importance of comprehensive histopathological evaluation of surgically resected fallopian tubes, even when removed for benign reproductive indications, and highlights the complex decision-making process regarding genetic counseling, surveillance strategies, and fertility preservation in reproductive-aged women with incidentally diagnosed STIC.

## Introduction

Serous tubal intraepithelial carcinoma (STIC) has emerged as a critical precursor lesion to high-grade serous ovarian carcinoma, the most lethal gynecologic malignancy with five-year survival rates below 50% [1]. This recognition has shifted understanding of ovarian cancer origins, with evidence supporting the fallopian tube as the primary site of malignant transformation [2]. STIC is characterized by nuclear pleomorphism, loss of cellular polarity, increased mitotic activity, and TP53 mutations [1,2]. Recent research has provided new insights into STIC pathogenesis, including the role of stromal microenvironment in malignant transformation [3]. While STIC is commonly identified during prophylactic salpingo-oophorectomy in BRCA carriers, with recent studies reporting incidence rates of 6.08% in BRCA-mutated women and 7.31% overall [1], sporadic cases in women without genetic predisposition are increasingly recognized. The incidental discovery of STIC during gynecological procedures poses clinical challenges, particularly in reproductive-aged women seeking fertility treatment.

Hydrosalpinx is a well-established negative prognostic factor in assisted reproductive technology (ART) outcomes, with meta-analyses demonstrating significantly reduced implantation rates, necessitating surgical intervention prior to embryo transfer [4]. However, histopathological examination of tubes removed for benign indications may reveal unexpected findings with profound clinical implications.

## Case presentation

A 43-year-old nulligravid Japanese woman presented with four years of primary infertility. Her medical history was unremarkable, and her family history included one maternal aunt with breast cancer, as well as gastrointestinal and liver cancers in grandparents, but no compelling hereditary pattern. Previous treatment included three oocyte retrievals and two embryo transfers without success.

Our fertility evaluation revealed normal hormone levels, with an anti-Müllerian hormone (AMH) level of 0.68 ng/mL (reference range: 0.72-1.67 ng/mL). At our clinic, she underwent nine additional oocyte retrievals over two years. Three subsequent embryo transfers were performed with optimal endometrial preparation, yet implantation failure persisted. Semen analyses consistently showed normal parameters, excluding male factor infertility (Table 1).

**Table 1:** Summary of patient characteristics and assisted reproductive technology (ART) history. AMH: anti-Müllerian hormone

Category	Characteristic	Findings	Notes
Patient background	Age (at presentation)	43 years old	-
	Infertility duration	4 years	Primary infertility
	Medical history	Unremarkable	-
	Family history	Maternal aunt with breast cancer	No compelling hereditary pattern identified
Initial fertility evaluation	AMH level	0.68 ng/mL	Below the lower limit of the local reference range (0.72-1.67 ng/mL)
	Semen analysis	Normal parameters	Male factor infertility excluded
	Tubal status (pre-op)	Left hydrosalpinx (1.8 cm) identified on ultrasound	-
ART treatment history	Prior clinic	-	-
	Oocyte retrieval (OR) cycles	3 cycles	-
	Embryo transfer (ET) cycles	2 cycles	Unsuccessful
	Current clinic	-	-
	Additional OR cycles	9 cycles	Performed for over two years
	Additional ET cycles	3 cycles	Performed with optimal endometrial preparation
	Outcome	Persistent implantation failure	-
Final intervention	Procedure	Laparoscopic left salpingectomy	Performed prior to the next planned embryo transfer

During preparation for the fourth retrieval cycle, ultrasonography first revealed an enlarged lumen of the left fallopian tube to 1.8 cm, consistent with hydrosalpinx. Given the association between hydrosalpinx and reduced implantation rates in ART [4], laparoscopic salpingectomy was recommended.

**Figure 1:**
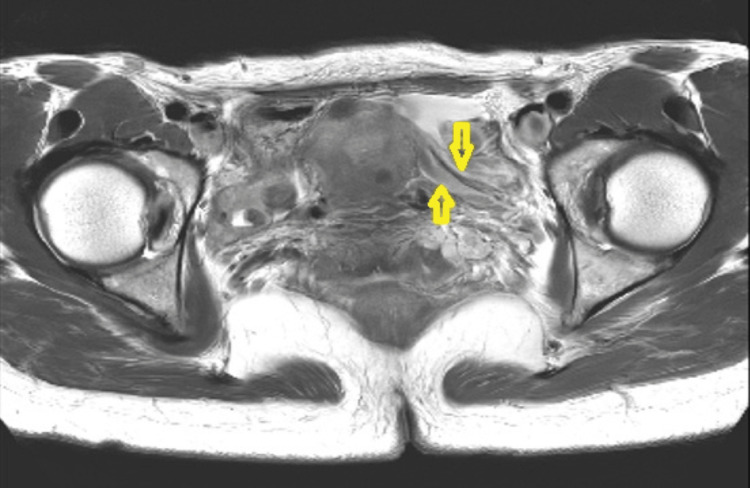
Magnetic resonance imaging (MRI), T2-weighted sequence demonstrating a swollen left fallopian tube (yellow arrows).

Preoperative evaluation included pelvic MRI, confirming left hydrosalpinx without other pathology (Figure 1).

**Figure 2:**
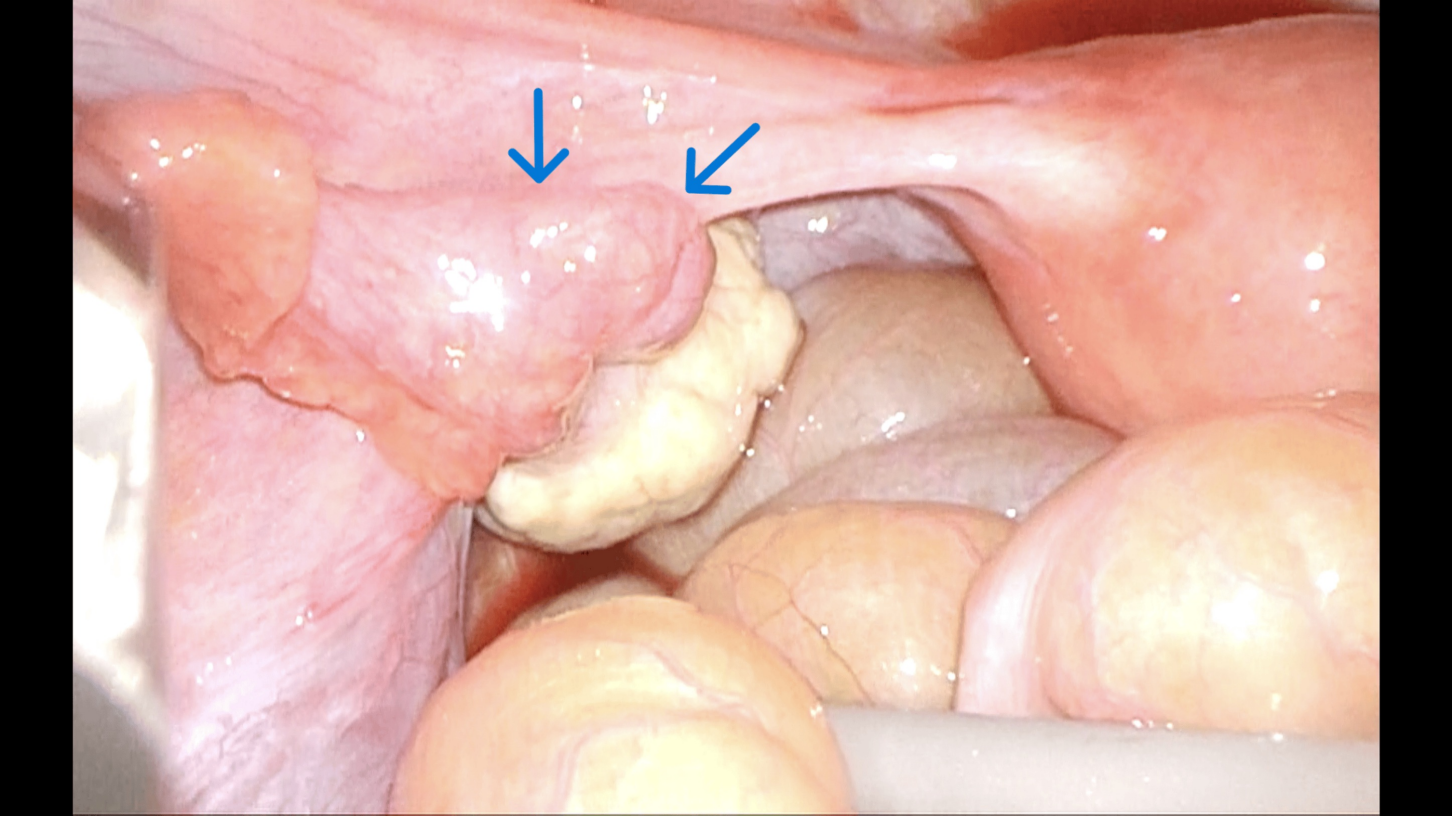
Laparoscopic findings showing swelling of the distal end of the left fallopian tube (blue arrows).

The patient provided informed consent for laparoscopic left salpingectomy with comprehensive histopathological examination. Laparoscopic left salpingectomy was performed using four 5 mm ports. The left ampulla of the fallopian tube was slightly enlarged (Figure 2).

**Figure 3:**
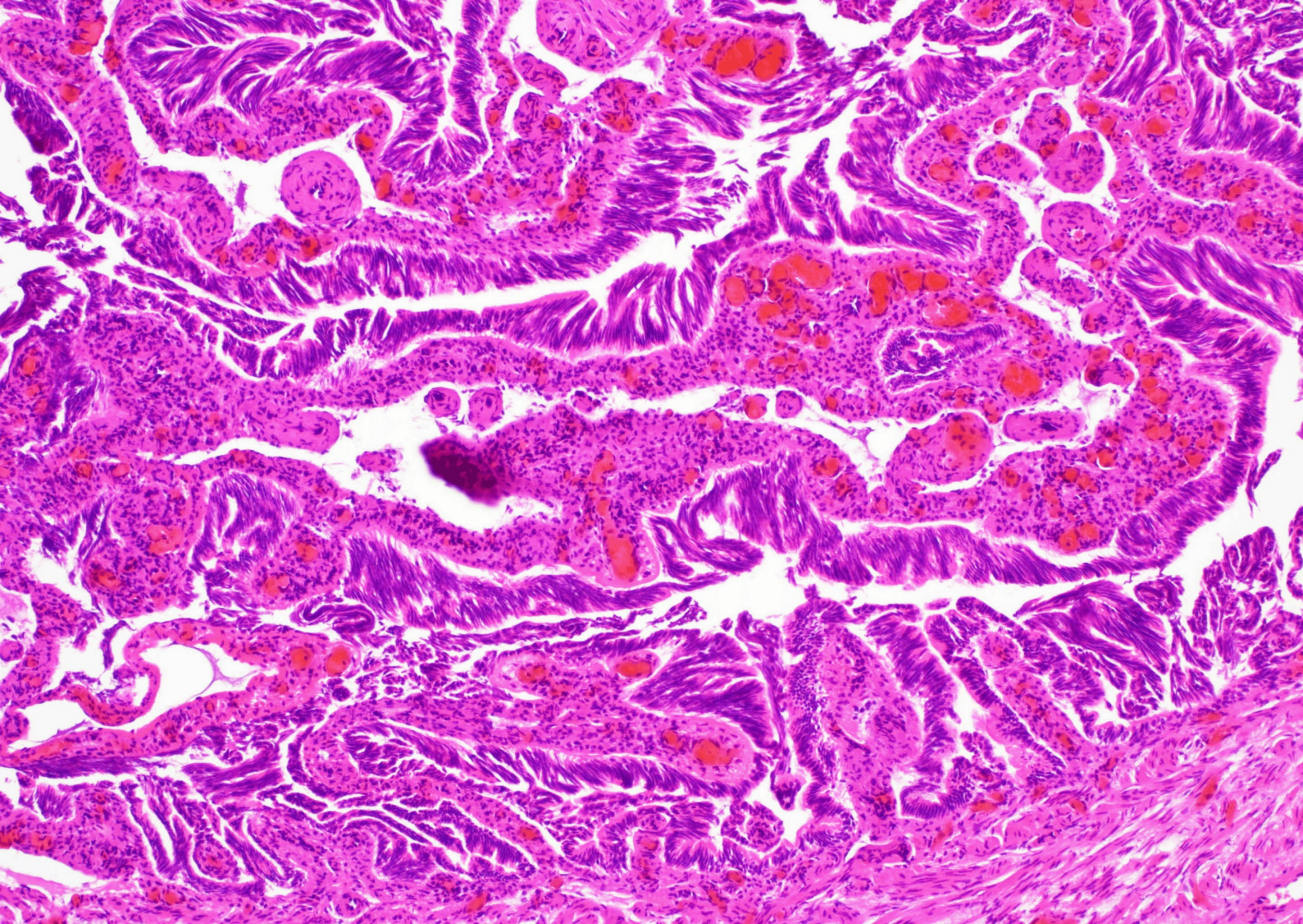
Hematoxylin and eosin-stained section of the fallopian tube epithelium (100× magnification).

The right tube appeared normal. The procedure was completed in 30 minutes without complications, and the patient was discharged on postoperative day 1. Histopathological examination revealed dysplastic glandular structures within the tubal epithelium, with architectural disarray and nuclear atypia. Hematoxylin and eosin staining confirmed stratified epithelium and loss of polarity. Immunohistochemistry demonstrated strong nuclear p53 expression and a Ki-67 index of 15%, along with positivity for CK7, EMA, ER, and WT-1; napsin A was negative. These findings supported the diagnosis of STIC (Figure 3).

Ascitic fluid cytology revealed atypical cells without malignant features. Postoperative CT imaging showed no evidence of metastatic disease. The patient was referred to gynecologic oncology for specialized counseling regarding an incidental STIC diagnosis.

Genetic counseling was offered to discuss BRCA testing. However, the patient declined testing, concerned that positive results might necessitate prophylactic bilateral salpingo-oophorectomy, compromising fertility goals. Following multidisciplinary consultation involving reproductive endocrinology, gynecologic oncology, and genetic counseling teams, surveillance was implemented.

The surveillance protocol included clinical examination every three months, ultrasonography and tumor marker assessment every six months, and annual pelvic MRI. The patient resumed ART cycles under enhanced surveillance. At six-month follow-up, she remained asymptomatic with stable imaging, and fertility treatment continued without disease progression.

## Discussion

Incidental diagnosis of STIC during ART is extremely rare, highlighting the intersection of reproductive medicine and gynecologic oncology. STIC is now established as the predominant precursor to high-grade serous carcinoma, with molecular characteristics closely paralleling invasive disease, including TP53 mutations and chromosomal instability [1,2].

The incidental discovery of STIC in our patient, who had no family history suggestive of hereditary cancer syndrome, underscores the importance of comprehensive pathological evaluation even in routine reproductive procedures. This finding challenges the traditional paradigm that STIC is primarily associated with hereditary cancer syndromes and emphasizes that sporadic cases can occur in women undergoing fertility treatment.

This case strongly supports the critical importance of rigorous histopathological examination of all surgically resected fallopian tubes, regardless of the indication for removal. The implementation of the Sectioning and Extensively Examining the Fimbria (SEE-FIM) protocol is crucial, as systematic reviews confirm its use is essential for maximizing the detection of STIC and thus informing clinical decision-making, even in cases of benign tubal disease like hydrosalpinx [5].

The intersection of STIC diagnosis with active fertility treatment presents unprecedented challenges requiring individualized, multidisciplinary decision-making that balances oncological risk with reproductive aspirations. Current evidence suggests STIC lesions may remain stable for extended periods, with progression to invasive carcinoma occurring over years to decades, though the natural history in reproductive-aged women pursuing fertility treatment remains poorly characterized [2,6].

The role of genetic testing in incidental STIC warrants careful consideration within the context of reproductive goals. While BRCA mutations significantly increase STIC prevalence, most sporadic cases occur without an identifiable genetic predisposition [7]. Testing decisions should be individualized based on family history, patient preferences, and reproductive goals, as demonstrated in our case, where the patient prioritized fertility preservation over genetic risk assessment. While the patient declined genetic testing, it must be noted that studies on isolated STIC demonstrate that the subsequent development of peritoneal carcinoma is almost exclusively observed in BRCA1 carriers [8]. Therefore, her decision introduced a necessary, yet calculated, long-term risk of relying solely on surveillance while actively pursuing fertility treatment.

This case underscores the critical importance of comprehensive histopathological examination of all surgically resected fallopian tubes, regardless of indication for removal. Implementation of standardized protocols for tubal examination, including the SEE-FIM protocol with extensive sectioning and microscopic evaluation, may facilitate early detection of precursor lesions [9,10].

The optimal surveillance strategy for reproductive-aged women with incidentally discovered STIC remains undefined, representing an urgent area for clinical research. Current recommendations extrapolated from BRCA carrier management include periodic imaging, tumor marker assessment, and clinical evaluation, though evidence-based guidelines specific to this population are critically needed [9,11].

The intersection of STIC diagnosis with active fertility treatment presents unprecedented challenges requiring individualized, multidisciplinary decision-making that balances oncological risk with reproductive aspirations. Current evidence suggests STIC lesions may remain stable for extended periods, with progression to invasive carcinoma occurring over years to decades, though the natural history in reproductive-aged women pursuing fertility treatment remains poorly characterized [2,6]. The optimal management of isolated STIC, particularly in women prioritizing fertility preservation, remains highly debated due to the rarity of this incidental finding. Recent multicenter data reveal significant variations in clinical management, highlighting the lack of a standardized protocol. Studies suggest the five-year cumulative incidence of high-grade serous carcinoma after an isolated STIC diagnosis is approximately 5.7% [9]. This emphasizes the necessity of the individualized, enhanced surveillance protocol implemented in our case, which includes tumor marker assessment and regular imaging.

## Conclusions

To our knowledge, this is the first case report in which STIC was incidentally detected after removal of hydrosalpinx as a part of infertility treatment. While removal of hydrosalpinx at reproductive age should be considered, optimal management of fertility preservation after diagnosis of STIC remains a topic for future investigation.
